# Supplementary material for: Close Association between Awareness of Teeth-Alignment Disorder and Systemic Disorders in Late Adolescence
Source: Healthcare (Basel). 2021 Mar 26;9(4):370. doi: 10.3390/healthcare9040370 (PMC8066092; doi:10.3390/healthcare9040370)
Supplement: Supplementary file 1 [file healthcare-09-00370-s001.pdf]

healthcare

MDPI

Table S1. Awareness of Teeth-Aligment Disorder.

"Are you concerned about alignment of your teeth (appearance of teeth alignment)?"	Yes	No
	□	□

Medical History

Please choose the presence or absence of the following disease(s)/disorder(s). When you chose the presence of a history for diseases/disorders, please write the specific name(s) of the disease(s)/disorder(s).

		Presence	Absence	Name of disease(s)/disorder(s)
**1**	Neuromasucular disease	□	□	
**2**	Cerebrovascular disease	□	□	
**3**	Coronary artery disease	□	□	
**4**	The other vascular disease	□	□	
**5**	Heart disease, arrhythmia, or abnormal electrocardiogram	□	□	
**6**	Hypertention	□	□	
**7**	Respiratory disease	□	□	
**8**	Esophagus, gastrointestinal disease	□	□	
**9**	Liver, gall bladder, pancreatic disease	□	□	
**10**	Renal disease	□	□	
**11**	Hyperuricemia, gout	□	□	
**12**	Diabetes mellitus	□	□	
**13**	Dyslipidemia	□	□	
**14**	Thyroid disease	□	□	
**15**	Endocrine disease	□	□	
**16**	Collagen disease	□	□	
**17**	Blood disease	□	□	
**18**	Malignant neoplasm	□	□	
**19**	Allergic disease	□	□	
**20**	Eye disease	□	□	
**21**	Otorhinolaryngologic disease	□	□	
**22**	Skin disease	□	□	
**23**	Bone, joint, muscle diseases	□	□	
**24**	Urologic disease, gynecological disease	□	□	
**25**	The other	□	□	
